# Royal College of Physicians, London

**Published:** 1852-11

**Authors:** 


### ROYAL COLLEGE OF PHYSICIANS, LONDON.

*Pall-Mall East, Trafalgar-square.*

*President*—Dr. John Ayrton Paris.

*Censors*—Drs. Todd, Crawford, Webster, and Owen Rees.

*Elects*—Drs. Thomas Turner, Clement Hue, John Bright, Edward Thomas Monro, Thomas Mayo, Henry Herbert Southey, Francis Hawkins.

*Treasurer*—Dr. Edward Thomas Monro.

*Registrar*—Dr. Francis Hawkins.

*The Examiners for the Licence are the President and Censors.*—The examinations take place on or about Christmas, Easter, Midsummer, and Michaelmas. The Examiners for the Extra-Licence are the President and three Elects. The examinations for the Extra-Licence take place at the same time as the former.

Fees—*Licence*, £56 17*s.*, including £15 stamp. *Fellowship*, £55 1*s.*, including £25 stamp. *Extra-Licence*, about £25.

*Regulations, dated* 1838, *and now in force.*

Every candidate for a licence or extra-licence must produce evidence,

1. Of unimpeached moral character.
2. Of having completed the twenty-sixth year of his age.

3. Of having devoted himself for five years, at least, to the study of medicine.

The course of study thus ordered by the College comprises:—

Anatomy and Physiology; the Theory and Practice of Physic; Forensic Medicine; Materia Medica and Botany; and the Principles of Midwifery and Surgery.

Attendance for three entire years on the physicians' practice of some general hospital or hospitals in Great Britain or Ireland, containing at least 100 beds, and having a regular establishment of physicians as well as surgeons.

Candidates who have been educated abroad, in addition to the full course of study specified, must have diligently attended the physicians' practice in some general hospital in this country, for at least twelve months.

Candidates who have already been engaged in practice, and have attained the age of forty years, but have not passed through the complete course of study above described, may be admitted to examination upon presenting to the Censors' Board such testimonials of character, general and professional, as shall be satisfactory to the college.

The first examination is in anatomy and physiology; the second includes all that relates to the causes and symptoms of diseases; the third relates to the treatment of diseases.

The examinations are carried on usually during three days by writing, and two days *viva voce*. The *viva voce* part of each is carried on in Latin, except when the Board deems it expedient to put questions in English, and permits answers to be returned in the same language.

A competent knowledge of Greek is recommended, but is not indispensable, if the other qualifications of the candidate prove satisfactory. The candidate is called on to translate *viva voce* into Latin a passage from Hippocrates, Galen, Aretæus; or to construe into English a portion of the works of Celsus, or Sydenham, or some other Latin medical author, having been previously required, on three separate days, to give written answers in English to questions on the different subjects enumerated above, and to translate, in writing, passages from Greek or Latin books relating to medicine.

The fellows are elected from the body of licentiates—a certain number generally on the 25th of June.

